# The Accuracy of a Commercial Wearable Neonatal Home‐Monitoring Device: A Simulation Study

**DOI:** 10.1111/jpc.70471

**Published:** 2026-06-15

**Authors:** Amelia N. Noone, Danielle N. Bailey, Tara M. Crawford, Shem Richards, Chad C. Andersen, Michael J. Stark

**Affiliations:** ^1^ Robinson Research Institute, College of Health, Adelaide University Adelaide South Australia Australia; ^2^ Goldilocks Suit Adelaide South Australia Australia; ^3^ Department of Neonatal Medicine Women’s and Children’s Hospital Adelaide South Australia Australia

**Keywords:** accelerometry, apnoea, infant, mannequin, simulation, wearable electronic device

## Abstract

**Objective:**

Wearable newborn‐monitoring devices are increasingly popular and widely available for home use. One approach includes the use of accelerometer detection of breathing movements. We investigated the accuracy of breathing and apnoea detection by a wearable accelerometer in a simulated environment.

**Design:**

This study used simulation as a method to evaluate the performance of a wearable accelerometer‐based breathing detection device. A term mannequin fitted with an accelerometer suit wirelessly linked to a mobile app (Goldilocks) was NeoPuff ventilated through a cuffed endotracheal tube. Peak inspiratory pressure (PIP) levels of 5–25 cmH_2_O were delivered with zero end expiratory pressure. Each trial consisted of 30 s of ventilation, followed by simulated apnoea (*n* = 150). App‐based detection and alert times were recorded blinded to ventilation status. Detection accuracy across PIP levels was analyzed using Kruskal–Wallis tests followed by Dunn's multiple comparisons with Bonferroni correction.

**Results:**

PIP level affected both apnoea detection (H(4) = 58.9, *p* < 0.0001) and breathing detection (H(4) = 86.5, *p* < 0.001) with performance reduced at 5 cmH_2_O compared to higher PIP. Apnoea and breathing detection were most accurate at 20 cmH_2_O. Mean (SD) apnoea detection alarm times ranged between 31(0) and 60(22.6) seconds, with no difference between PIP group (H(4) = 9.3, *p* > 0.05).

**Conclusions:**

The accelerometer displayed pressure‐dependent accuracy, increasing with higher PIP, suggesting a minimum pressure threshold is needed for reliable monitoring. App alert times varied greatly, compounding the accuracy of adverse event detection. To determine their true utility, rigorous investigation of neonatal wearable home‐monitoring devices is required.

## Key Points

- What is already known on this topic
  ○ There is currently limited evidence to support the accuracy or clinical value of newborn‐monitoring devices in detecting breathing or apnoea events in the home environment.
- What this study adds
  ○ Accelerometer‐based newborn‐monitoring devices may exhibit pressure‐dependent accuracy in detecting breathing and/or apnoea.
  ○ Apnoea monitor alarms may be delayed from the actual apnoeic event.

## 1 Introduction

Wearable monitoring devices for newborns are becoming increasingly popular and widely available for home use. These devices promote parental ease of mind and assist in tracking infant vitals in the home environment. Wearable technology sensors for neonates are available in various designs, including those integrated into clothing, socks and mattresses. Physiological measures including heart rate, oxygen saturation and respiration can be measured by these devices [1]. Parents can access the health information of their infant through an integrated smartphone app and receive alerts when the device detects physiological abnormalities. These non‐invasive devices promote remote monitoring of newborns and can assist with parental anxiety. As a result, there has been a dramatic increase in commercially available wearable single‐ and multiple‐parameter technologies focusing on infant health monitoring in the last 5 years [2]. However, there is a notable absence of evidence‐based recommendations for the use of these devices. As a result, smartphone‐integrated consumer infant monitors that measure vital signs are not policed by Federal regulatory bodies [3, 4]. Indeed, routine home infant monitor use is not recommended by The American Academy of Paediatrics due to a lack of evidence for the prevention of morbidity or mortality [5].

There are currently numerous neonatal home‐monitoring devices available on the market. The Goldilocks Suit [6] is a smart singlet that records temperature, feeding, sleeping and breathing. Core and skin temperature sensors are screen‐printed into the fabric of the singlet, while breathing waveforms are captured using an accelerometer in a pocket of the singlet positioned over the lower chest and diaphragm. This device is wireless, allowing ease of parent and infant bonding, feeding, movement and daily care. Upon detection of respiratory abnormalities, parents are alerted on a smartphone app using a traffic light system with a green icon signifying normal breathing, an orange icon indicating the detection of abnormalities and a red icon to signal the detection of apnoea.

In order to contribute to the limited body of evidence surrounding neonatal wearable monitoring devices and inform future recommendations for their home use, this study aimed to evaluate the accuracy and reliability of this accelerometer‐based approach in detecting breathing and apnoea events in a simulated environment using an infant resuscitation mannequin.

## 2 Methods

### 2.1 Device Information

The Goldilocks neonatal wearable monitoring device uses a 30‐s window to capture data which updates the breathing status according to the algorithm. Breathing waveforms are detected by an accelerometer through the identification of high and low points. These are converted to a breathing or apnoea score within the algorithm, which then updates the parental interface app. To attain a ‘breathing’ score, there must be more than 10 high and low points in the 30‐s window to capture a breathing waveform. Infant core temperature must be above 30°C for the mobile phone app to detect and alarm breathing status. If temperature is over or under the range, the red alarm will appear and cannot re‐alarm for apnoea detection until temperature range is maintained for another 30‐s period. These algorithmic‐dependent features overall contribute to delayed apnoea detection in the device, and subsequent delayed alarm. To ensure that this was prevented, the experiment was conducted within an isolette with a set temperate of 36.5°C.

### 2.2 Experimental Design

A standard term resuscitation mannequin wearing an accelerometer‐containing suit, connected wirelessly to a mobile phone app (Goldilocks), was NeoPuff ventilated via a cuffed endotracheal tube in a supine position. The accelerometer sat inside a fitted pocket on the singlet above the left diaphragm. All repeated standardized ventilation trials were conducted in a clinical environment within a set temperature isolette to enhance measurement reliability. To determine baseline ventilation, the mannequin received 40 breaths per minute, following a 30‐s apnoea period. Peak inspiratory pressure (PIP) was delivered at 5, 10, 15, 20 and 25 cmH_2_O, with zero end expiratory pressure. This sequence was repeated 30 times for each PIP. Blinded to ventilation status, the time until a red alarm appeared on the smartphone‐integrated app was recorded. The researcher controlling airflow to the mannequin was blinded to the outcome on the app. Raw timestamped data was collected from the accelerometer and analyzed independently, blinded to ventilation status to reduce measurement bias. The manuscript was prepared in accordance with the SQUIRE‐SIM guideline (Table 1) [7].

**TABLE 1:** SQUIRE‐SIM extension guidelines.

Text section and item name	SQUIRE 2.0 item	SQUIRE‐SIM extension	Included	Line number
Notes to authors	The SQUIRE guidelines provide a framework for reporting new knowledge about how to improve healthcare. The SQUIRE guidelines are intended for reports that describe system level work to improve the quality, safety and value of healthcare, and used methods to establish that observed outcomes were due to the intervention(s). A range of approaches exists for improving healthcare. SQUIRE may be adapted for reporting any of these. Authors should consider every SQUIRE item, but it may be inappropriate or unnecessary to include every SQUIRE element in a particular manuscript. The SQUIRE glossary contains definitions of many of the key words in SQUIRE. The Explanation and Elaboration documents provide specific examples of well‐written SQUIRE items, and an in‐depth explanation of each item. Please cite SQUIRE when it is used to write a manuscript.	The SQUIRE SIM extension focuses on strategies for reporting simulation‐based studies intended to improve healthcare education and/or delivery where simulation is used as an intervention (simulation used to make a change) or method of study (simulation to evaluate a change).For those studies in which simulation is used as an educational intervention, aspects of SQUIRE‐EDU may also be applicable.Authors should consider every SQUIRE and SQUIRE‐SIM item, but it may be unnecessary to include every SQUIRE or SQUIRE‐SIM element in a particular manuscript. Emphasize “simulation as an intervention” in the intervention sections (#8 & #9), and “simulation as the method of study” in measures (#10) and analysis (#11). Although some information may be relevant in multiple sections of the manuscript, select the optimal section and avoid repetition.Please cite SQUIRE‐SIM when it is used to write a manuscript.These guidelines may also be useful as a framework when designing a simulation‐based quality improvement project to ensure all suggested elements have been met.		
Title and abstract				
1. Title	Indicate that the manuscript concerns an initiative to improve healthcare (broadly defined to include the quality, safety, effectiveness, patient‐centredness, timeliness, cost, efficiency and equity of healthcare).	**SIM 1**: Indicate that the manuscript concerns a simulation‐based quality improvement initiative.	Yes	1–2
2. Abstract	a. Provide adequate information to aid in searching and indexing.	**SIM 2a**: Keywords include “simulation,” “simulated,” or “simulation‐based”	Yes	8–9
	b. Summarize all key information from various sections of the text using the abstract format of the intended publication or a structured summary such as: background, local problem, methods, interventions, results, conclusions	**SIM 2b**: Include how simulation is used in the study as an intervention, a QI method of study, or both.	Yes	9–10
Introduction: Why did you start?				
3. Problem description	Name and significance of the local problem.	___		
4. Available knowledge	Summary of what is currently known about the problem, including relevant previous studies.	**SIM 4**: Include relevant studies in which simulation was used in the literature review.	N/A	
5. Rationale	Informal or formal frameworks, models, concepts and/or theories used to explain the problem, any reasons or assumptions that were used to develop the intervention(s) and reasons why the intervention(s) was expected to work.	**SIM 5:** Include relevant simulation‐specific frameworks, theories or experiences, if available, that guided the choice and implementation of simulation for the current problem.	N/A	
6. Specific aims	Purpose of the project and of this report.		Yes	71–75
Methods: What did you do?				
7. Context	Contextual elements considered important at the outset of introducing the intervention(s).	**SIM 7:** Include the contextual elements for simulation‐based quality improvement projects and simulation team characteristics including enough detail of the simulation‐specific aspects of the intervention for reproducibility. See Figure 1 for further details and examples.	Yes	91–104
8. Intervention(s)	a. Description of the intervention(s) in sufficient detail that others could reproduce it.			
	b. Specifics of the team involved in the work.			
9. Study of the intervention(s)	a. Approach chosen for assessing the impact of the intervention(s)	**SIM 9a:** When simulation is the intervention, describe any iterative changes to the simulation itself or means to standardize simulation delivery.	N/A	
10. Measures	b. Approach used to establish whether the observed outcomes were due to the intervention.	**SIM 9b**: When simulation is the intervention, describe how variability across simulations was managed.	N/A	
	a. Measures chosen for studying processes and outcomes of the intervention(s), including rationale for choosing them, their operational definitions and their validity and reliability.	**SIM 10a**: When simulation is used as the method of study, describe how simulation was used to assess the outcome of the intervention and the means used to enhance and assess the validity and reliability of the measures, e.g., rater training, comparison of independent raters.	Yes	91–104
	b. Description of the approach to the ongoing assessment of contextual elements that contributed to the success, failure, efficiency and cost.	**SIM 10b**: When simulation is the method of study, describe the approach used to assess simulation‐specific contextual elements, such as realism, simulation setting/modality, generalizability, that contributed to the success, failure, efficiency and cost.	Yes	81–89, 94–96, 100–102
	c. Methods employed for assessing completeness and accuracy of data	**SIM 10c**: When simulation is the method of study, describe the methods and means of data acquisition, such as use of checklists, sensors embedded within the simulators, real‐time observations, video review, tracking devices, debrief analysis or other means.	Yes	91–104
11. Analysis	a. Qualitative and quantitative methods used to draw inferences from the data	__	N/A	
	b. Methods for understanding variation within the data, including the effects of time as a variable.	**SIM 11b**: When simulation is the intervention, describe the methods for assessing consistency across simulations. When simulation is the method of study, describe methods for assessing outcomes related to condensed time intervals.	N/A	
12. Ethical considerations	Ethical aspects of implementing and studying the intervention(s) and how they were addressed, including, but not limited to, formal ethics review and potential conflict(s) of interest.	**SIM 12:** Describe approaches to mitigate potential risks from simulation such as patient and staff physical safety during an in situ simulation, efforts to maintain psychological safety and confidentiality, especially as it relates to relationship of participants and observers.	N/A	
Results: What did you find?				
13. Results	a. Initial steps of the intervention(s) and their evolution over time (e.g., time‐line diagram, flow chart or table), including modifications made to the intervention during the project.	**SIM 13a:** Describe any unintended deviations or iterative changes to the simulation and the impact of these changes on the results.	N/A	
	b. Details of the process measures and outcomes.	**__**		
	c. Contextual elements that interacted with the intervention(s).	__		
	d. Observed associations between outcomes, intervention(s) and relevant contextual elements.			
	e. Unintended consequences such as unexpected benefits, problems, failures or costs associated with the intervention(s).	**SIM 13e**: Describe any unintended consequences, unexpected benefits or safety issues that may have resulted from the simulation‐based elements of the study.	N/A	
	f. Details about missing data.	__		
Discussion: What does it mean?				
14. Summary	a. Key findings, including relevance to the rationale and specific aims.	__		
	b. Particular strengths of the project.	**SIM 14b:** Include the strength of simulation as intervention or method of study as compared to other methods of study, i.e., why might have simulation worked better than other methods?	Yes	224–227, 233–234
15. Interpretation	a. Nature of the association between the intervention(s) and the outcomes.	**SIM 15:** Describe the transferability of simulation‐based training to clinical performance. Describe the extent to which the simulation‐based intervention or outcomes can be generalized to patient‐based outcomes. Discuss cost effectiveness, if applicable.	Yes	220–235
	b. Comparison of results with findings from other publications.			
	c. Impact of the project on people and systems.			
	d. Reasons for any differences between observed and anticipated outcomes, including the influence of context.			
	e. Costs and strategic trade‐offs, including opportunity costs.			
16. Limitations	a. Limits to the generalizability of the work.	**SIM 16a:** Specify the simulation‐specific limitations that may limit generalizability such as realism, location, learner acceptance, participant population.	Yes	230–233, 236–237
	b. Factors that might have limited internal validity such as confounding, bias or imprecision in the design, methods, measurement or analysis.	**SIM 16b:** Specify simulation‐specific factors that may have limited validity.	Yes	230–233
	c. Efforts made to minimize and adjust for limitations.	__	N/A	
17. Conclusions	a. Usefulness of the work.	__	Yes	254–256
	b. Sustainability.	__	N/A	
	c. Potential for spread to other contexts.	**SIM 17c**: Describe the scalability and transferability of the simulation intervention or assessment to other individuals, teams, systems and to real patients or situations.	Yes	220–233
	d. Implications for practice and for further study in the field.	**SIM 17d:** If simulation is the intervention, include lessons learned for clinical practice, care experience, education, training and policy. If simulation is the method of study, include implications of this assessment process for future studies.	Yes	261–266
	e. Suggested next steps.	__	Yes	265–266
18. Funding	Sources of funding that supported this work. Role, if any, of the funding organization in the design, implementation, interpretation and reporting.	__	N/A	

*Note:* This table provides the SQUIRE‐SIM extension guidelines that should be used for simulation‐based quality improvement projects. Examples of well‐written SQUIRE‐SIM items can be found in Appendix A and at http://squire‐statement.org/.

### 2.3 Statistical Analysis

Accuracy across the five levels of PIP (5, 10, 15, 20 and 25 cmH_2_O) and time to apnoea detection was determined using the Kruskal–Wallis *H* test. Post hoc analysis of pairwise differences in breathing detection was assessed by Dunn's post hoc tests to correct for multiple comparisons with the normality of the distribution of the time to apnoea detection data assessed with the Shapiro–Wilks test. Data were analyzed using SPSS version 28 (IBM Corporation, Armonk, New York, USA) and GraphPad Prism version 9 (Boston, Massachusetts, USA). A *p* value of < 0.05 was considered statistically significant.

## 3 Results

**FIGURE 1:**
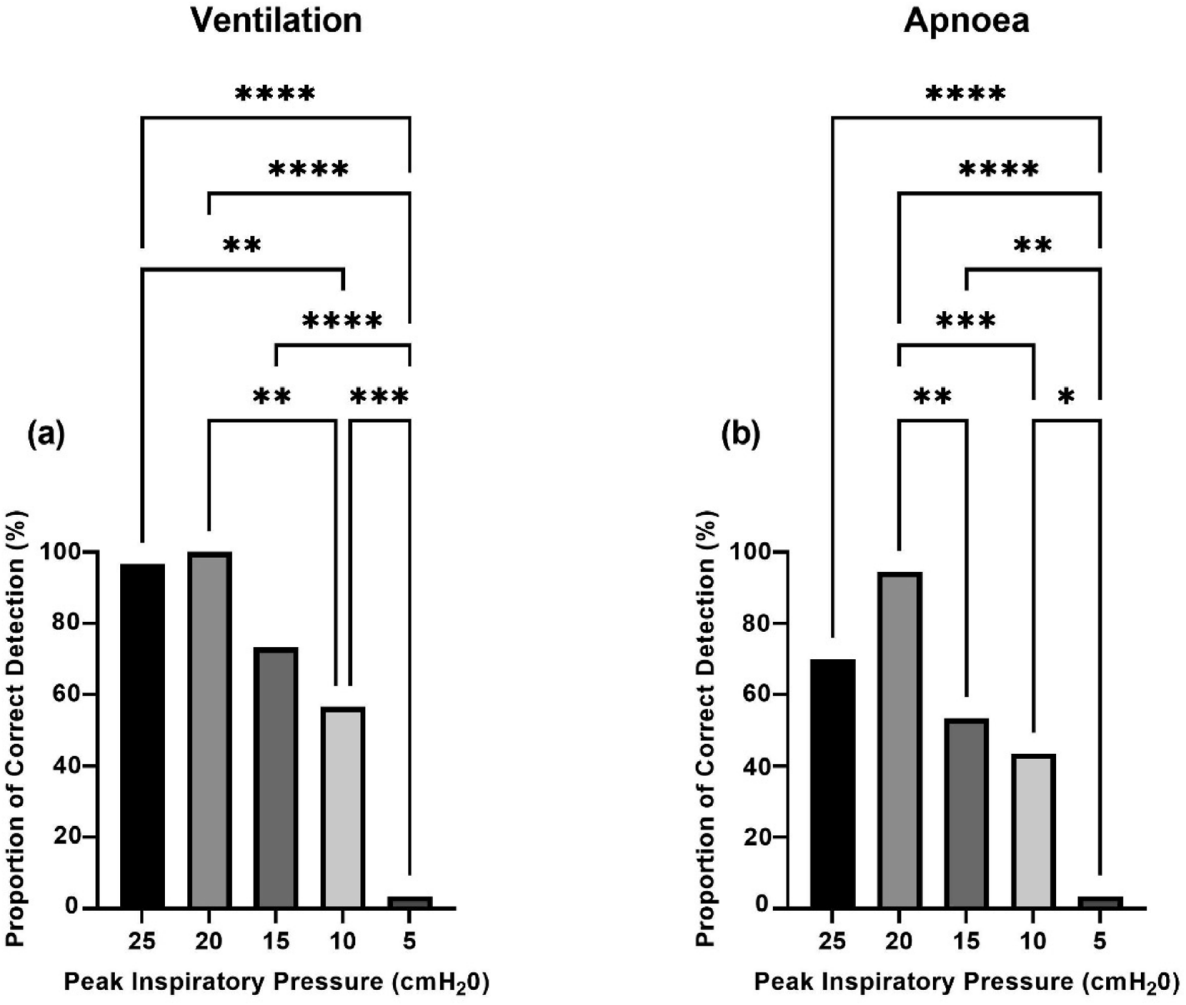
(a, b) Proportion of correct detection during ventilation (a) and apnoea trials (b). *p < 0.05, **p < 0.01, ***p < 0.001, ****p < 0.0001; ns , not significant (*p* ≥ 0.05).

With simulation as the method of study, breathing detection accuracy was lowest at 5 cmH_2_O and highest at 20 cmH_2_O, with significant differences across PIP levels, H(4) = 86.52, *p* < 0.001. Post hoc comparisons (Dunn's multiple comparisons test with Bonferroni correction) identified several significant differences (Figure 1a). 5 cmH_2_O resulted in significantly reduced breathing detection compared to all higher PIP: 10 cmH_2_O (*p* < 0.001), 15 cmH_2_O (*p* < 0.0001), 20 cmH_2_O (*p* < 0.0001) and 25 cmH_2_O (*p* < 0.0001). Significant differences were also found between 25 cmH_2_O and 10 cmH_2_O (*p* < 0.01), 20 cmH_2_O and 10 cmH_2_O (*p* < 0.01) and 15 cmH_2_O and 5 cmH_2_O (*p* < 0.001). No significant differences were found between the remaining levels of PIP.

Similarly, apnoea detection accuracy was lowest at 5 cmH_2_O and highest at 20 cmH_2_O, with significant differences across PIP groups, H(4) = 58.9, *p* < 0.001. When set at 5 cmH_2_O apnoea detection accuracy was significantly lower than at all higher PIP levels: 10 cmH_2_O (*p* < 0.05), 15 cmH_2_O (*p* < 0.01), 20 cmH_2_O (*p* < 0.0001) and 25 cmH_2_O (*p* < 0.0001) (Figure 1b). Differences were observed between 20 cmH_2_O and 15 cmH_2_O (*p* < 0.01), and 10 cmH_2_O (*p* < 0.01).

**FIGURE 2:**
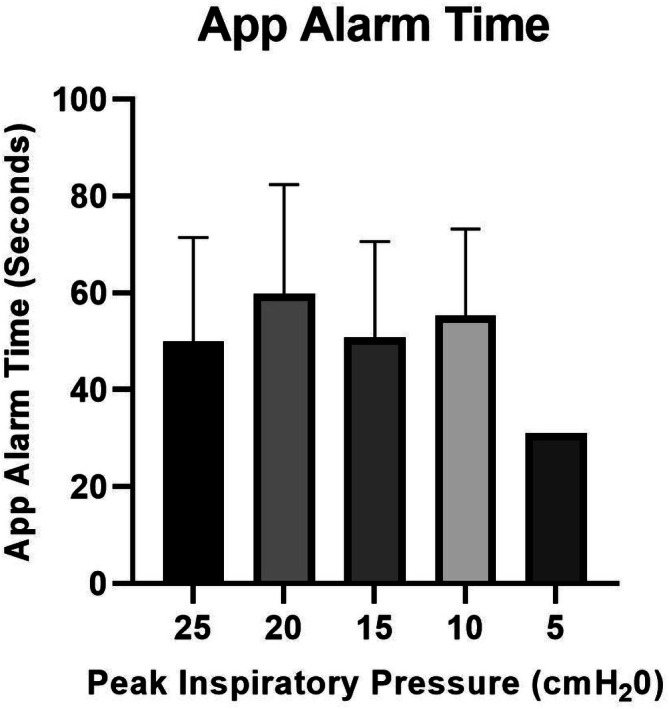
Time to apnoea detection on mobile phone app.

Blinded to ventilation status, the smartphone‐integrated app alarm time was recorded for each PIP trial (Figure 2). The average (SD) time to alarm on the app following apnoea ranged from 31 (0) to 60 (22.6) seconds. No significant difference was found in app alarm times between the PIP groups (H(4) = 9.35, *p* > 0.05).

## 4 Discussion

Although interest in neonatal home‐monitoring devices is growing, there is still a lack of strong evidence‐based recommendations to support their use. This study is the first to evaluate the accuracy of accelerometer‐based (Goldilocks) detection of simulated breathing and apnoea using an infant mannequin. Each PIP level can be applied repeatedly and identically, allowing multiple trials per condition. Further, varying the PIP resulted in a controllable, clinically meaningful, quantifiable ‘dose’ of chest movement allowing for rigorous, interpretable testing of the monitor's accuracy across its intended operating range. The device demonstrated optimal performance at a PIP of 20 cmH_2_O, where breathing was correctly detected in all trials and apnoea was subsequently identified in 94% of cases. At all other PIP levels, false negative readings occurred for both apnoea and breathing detection. Testing at lower PIP levels, which generate smaller chest displacements on the mannequin, directly validates whether the device has sufficient sensitivity for quiet, shallow breathing typical of a well‐baby at rest. Therefore, in the out‐of‐hospital well‐baby context, the multiple PIP levels are relevant because they model the range of normal and abnormal breathing effort that the device would need to monitor accurately in the real world. Detection accuracy declined with lower PIP, with only 3% of events correctly detected at 5 cmH_2_O. While the observed highly variable delays in the associated smartphone app alarm activation were unaffected by variations in PIP, substantial variability was observed, with alert times ranging from 30 to 82 s after the apnoea event. Taken together, these findings highlight pressure‐dependent accuracy and the potential risk of false parental reassurance when home monitors may fail to correctly identify clinically meaningful events [8].

Commercially available neonatal home‐monitoring devices clearly highlight that their products are not medical devices. As such, the purpose of these devices is parental ‘peace of mind’ and to provide insights into a baby's ‘wellness’ [6, 9], with manufacturers careful to stress that these technologies are not intended to prevent sudden infant death syndrome (SIDS) or to replace safe sleep practices [6, 9]. A systematic review by Strehle and colleagues concluded an absence of high‐level evidence regarding the effectiveness of home‐monitoring devices in the prevention of SIDS [10]. However, some authors have suggested that when used as an adjunct to a program with clinically based strategies, neonatal home‐monitoring devices can be an effective tool in infants at an increased risk of SIDS [11]. This apparently contradictory messaging in the medical literature can induce similar contradictory responses in parents [10]. Some reports have concluded that neonatal home‐monitoring devices as a whole ‘may cause unnecessary fear, uncertainty, and self‐doubt in parents’ [3, 11]. Yet, studies admittedly with declared links to home‐monitoring device manufacturers report high rates of parent satisfaction with up to 84% of parents reporting improved sleep quality for themselves and 96% reporting decreased anxiety despite risks of overdiagnosis and costs associated with these devices [4].

Our evaluation showed that the smartphone apnoea alert exhibited highly varied latencies, exceeding 1 min in some cases when compared to the corresponding clinical event. Although parents often interpret these alerts as real‐time indicators, this assumption is not supported by our data and has important implications for caregiver understanding and response. While a significant number of false negative readings were observed for both apnoea and breathing detection in the current study, constant monitoring will also unavoidably result in false positive alarms. These can desensitize parent responses, negatively influencing parental compliance [12]. Both false positive and true positive alarms may trigger further investigation by health care professionals, including emergency department visits [3]. Critically, due to the lack of evidence‐based knowledge surrounding these devices, the ability of caregivers, clinicians and healthcare professionals to meaningfully interpret the significance of the data leading to these clinical interactions remains limited.

The utility of wearable home‐monitoring devices to contribute to the early detection and diagnosis of health conditions remains a knowledge gap and has not been tested in any randomized controlled trials [8]. A single, randomized controlled trial of home monitoring conducted nearly 40 years ago enrolled 100 infants at higher risk of SIDS due to family history [13]. Infants in the intervention group were given an apnoea monitor, and both groups had weekly nurse visits. Parents of monitored infants reported 11% fewer symptoms, but there were no differences between groups in paediatric visits or hospital admissions, and no deaths occurred in either group. With respect to wearable home monitoring, a single study investigating the ability of the Owlet Smart Sock to aid in detection of clinically significant diagnoses reported outcomes for 47 495 infants who were monitored for 6 months over a 2‐year period [4]. A total of 49 parent‐reported cases were subsequently classified as clinically significant [4]. However, the number of false positive alarms that led to unnecessary healthcare investigation and intervention was not reported. There is further need for rigorous controlled studies to guide safe and effective use of neonatal monitoring devices in the home.

A comparison of the accuracy of the Goldilocks accelerometers' ability to detect breathing and apnoea against hospital standard monitoring was beyond the remit of the current study as we aimed to simulate its use in the home environment. Despite advancements in technology, the accuracy of these devices remains poorly understood, and there is limited clinical data for the majority of the commercially available wearable home devices. The accuracy of the Owlet Smart Sock has been tested against various hospital‐issued pulse oximetry devices. Compared to a FDA‐approved Masimo pulse oximeter, heart rate and SpO_2_ data were strongly correlated in 26 preterm or < 2.5 kg infants [14]. A more recent study comparing the Owlet Smart Sock 3.0 to standard hospital pulse oximetry in 66 preterm to term infants demonstrated high specificity for detecting bradycardia and hypoxaemia events with longer durations, indicating a low rate of false positives [15]. Despite this, there was a moderate correlation between the hospital‐issued SpO_2_ and Owlet Smart Sock SpO_2_ (*R* ^2^ = 0.48) [15]. However, in a 30‐infant comparison study of the Owlet Smart Sock 2 and Baby Vida, both commercially available home wearable monitoring devices, against the reference Masimo Radical‐7 monitor, 12 infants experienced hypoxemic episodes, with the Owlet Smart Sock incorrectly classifying at least one of these events as normoxemic in 5 out of the 12 cases [1]. Additionally, the Baby Vida monitor failed to correctly identify any hypoxemic events, whereas the reference monitor detected such events in 14 infants [1]. These inconsistencies raise concerns over their accuracy in detecting cardiorespiratory events, particularly for shorter events where the devices may show limited sensitivity.

This study had several limitations. The primary value of out‐of‐hospital monitoring in well babies is early detection of deterioration, the transition from normal breathing to something concerning. This will generally manifest as subtle changes in breathing depth and pattern before overt apnoea or distress develops, irrespective of the underlying pathological condition. By investigating whether the device can accurately discriminate between different chest wall excursion magnitudes across the PIP range tested, we can demonstrate the sensitivity and accuracy of an accelerometer‐based approach to detect early, subtle reductions in breathing effort. The primary aim of the study was not to address the technology's clinical application but instead demonstrate or otherwise the device's accuracy across physiologically relevant excursion ranges, thereby building evidence that the device is trustworthy as a standalone monitoring tool. The use of a simulation mannequin to facilitate controlled manipulation of ventilation is not reflective of organic breathing in a live neonate. As the wearable monitoring device detects breathing waveforms, simulated waveforms delivered by Neopuff may differ from organic breathing waveforms. However, the use of a mannequin was essential for simulating apnoea. Simulation enabled a low‐cost and efficient approach to assess device performance under controlled ventilation conditions.

A further limitation of this wearable device is its inability to detect obstructive apnoea. Obstructive apnoea is distinctive in that there is chest wall movement in the presence of partial or complete upper airway collapse, whereas central apnoea is defined by the absence of chest wall movement, as well as airflow. Due to the device configuration, breathing waveforms are based on accelerometer movements, the chest rise and fall of the infant. Obstructive apnoea includes chest wall movement; however, reduced or complete loss of airflow. The most common form of apnoea in infants is mixed apnoea, a combination of central apnoea typically followed by obstructive apnoea. This specific algorithm may not differentiate between obstructive apnoea and normal breathing patterns; however, this requires further investigation. In this device, alerts are delayed from real‐time events as the algorithm records data in 30‐s time blocks. For true alerts, a minimum of 10 high and low points of a breathing waveform must be detected over a 30‐s period. The variability observed in alarm times may be due to external factors, such as Wi‐Fi/connectivity delays or user‐related error. Given that the system's processing is computer‐driven, a coding issue appears unlikely, as this would be expected to produce consistent, rather than variable delays.

The current study represents the first assessment of an accelerometer‐based wearable home‐monitoring device to detect newborn breathing and apnoea in a simulated environment. The study essentially asks: ‘Can this device reliably detect and quantify chest wall movement across the full spectrum it will encounter in a well‐baby at home?’ Answering this fundamental question is a prerequisite for safe use. The detection of normal and abnormal breathing patterns was most accurate when PIP was set to 20 cmH_2_O. As PIP decreased, there was a corresponding significant decline in the accuracy of breathing detection, though no difference in the smartphone apnoea alarm time which showed significant variability across all ventilation PIP groups. These findings support a pressure‐dependent relationship in the device's ability to detect respiratory events. Wearable home‐monitoring devices have the potential to enhance the patient discharge process and outpatient monitoring abilities of neonates. However, commercially available neonatal monitoring devices for the home are not regulated to the same extent as medical devices. The lack of evidence‐based recommendations surrounding these devices highlights the need for rigorous investigation of the true utility of wearable monitoring devices in the home.

## Author Contributions

All authors contributed to the planning of the study design. A.N.N. and M.J.S. were involved in the literature search and preparation of the article. All authors contributed to the methodology and data collection. A.N.N. performed data analysis, and S.R. performed data analysis, blinded to intervention status. A.N.N. was involved in writing the original draft preparation, and all authors were involved in review and editing. All authors have read and agreed to the published version of the manuscript.

## Funding

The authors have nothing to report.

## Ethics Statement

The authors declare that the research presented in this manuscript adheres to the ethical principles outlined by the Women’s and Children’s Health Network. All procedures involving human participants were conducted in accordance with the ethical standards of The University of Adelaide and the Declaration of Helsinki (1964), as revised in 2013.

## Conflicts of Interest

S.R. is the founder of Goldilocks and supplied the Goldilocks Suit for research use and conducted blinded data analysis.

## Data Availability

The data that support the findings of this study are available from the corresponding author upon reasonable request.
